# Maternal dendrimer-based therapy for inflammation-induced preterm birth and perinatal brain injury

**DOI:** 10.1038/s41598-017-06113-2

**Published:** 2017-07-21

**Authors:** Jun Lei, Jason M. Rosenzweig, Manoj K. Mishra, Wael Alshehri, Flavia Brancusi, Mike McLane, Ahmad Almalki, Rudhab Bahabry, Hattan Arif, Rayyan Rozzah, Ghada Alyousif, Yahya Shabi, Nader Alhehaily, Wenyu Zhong, Andrea Facciabene, Sujatha Kannan, Rangaramanujam M. Kannan, Irina Burd

**Affiliations:** 1 Integrated Research Center for Fetal Medicine, Johns Hopkins University School of Medicine, Baltimore, MD USA; 2 Center for Nanomedicine, Johns Hopkins University School of Medicine, Baltimore, MD USA; 3 Department of Obstetrics and Gynecology, University of Pennsylvania, Philadelphia, PA USA; 4 Anesthesiology and Critical Care Medicine, Johns Hopkins University School of Medicine, Baltimore, MD USA; 5 Department of Neuroscience, Kennedy Krieger Institute, Baltimore, MD USA; 6 Department of Neurology, Johns Hopkins University School of Medicine, Baltimore, MD USA

## Abstract

Preterm birth is a major risk factor for adverse neurological outcomes in ex-preterm children, including motor, cognitive, and behavioral disabilities. *N*-acetyl-*L*-cysteine therapy has been used in clinical studies; however, it requires doses that cause significant side effects. In this study, we explore the effect of low dose *N*-acetyl-*L*-cysteine therapy, delivered using a targeted, systemic, maternal, dendrimer nanoparticle (DNAC), in a mouse model of intrauterine inflammation. Our results demonstrated that intraperitoneal maternal DNAC administration significantly reduced the preterm birth rate and altered placental immune profile with decreased CD8^+^ T-cell infiltration. Furthermore, we demonstrated that DNAC improved neurobehavioral outcomes and reduced fetal neuroinflammation and long-term microglial activation in offspring. Our study is the first to provide evidence for the role of CD8^+^ T-cell in the maternal-fetal interface during inflammation and further support the efficacy of DNAC in preventing preterm birth and prematurity-related outcomes.

## Introduction

Spontaneous preterm birth, a condition often associated with intrauterine inflammation, increases the risk of adverse neurologic and developmental outcomes such as cerebral palsy (CP), autism spectrum disorder, attention and behavior abnormalities, and motor or cognitive deficits in offspring^1–10^. The etiology of perinatal brain injury is complex and involves a multifaceted response to inflammation including glial activation, production of reactive oxygen species (ROS), oxidative stress and, ultimately, the loss of neurons^11–17^. Administration of antioxidants has been employed successfully in animal models of intrauterine inflammation to reduce perinatal inflammatory response^18, 19^.

*N*-acetyl-*L*-cysteine, or NAC, is a prodrug that increases the pool of available cysteine, a precursor to the antioxidant glutathione (GSH). NAC is also an antioxidant with anti-inflammatory properties and is used clinically for acetaminophen poisoning. Oral NAC administration can reduce rates of recurrent preterm labor in patients with a history of bacterial vaginosis^20^. However, side effects at high doses necessary for this intervention have led to problems with compliance. In animal models, NAC has been shown to attenuate expression of pro-inflammatory cytokines in macrophages, and also to protect against preterm labor, but at doses that are much higher than those used clinically^21^. NAC also reduced the immune response in rat fetal brains^22^.

While NAC can be effective at reducing inflammation-induced preterm birth, it has limitations. The effective doses for free NAC are high, and can include side-effects such as nausea, vomiting, stomatitis, and fever. Nanoparticle-based drug delivery systems offer many advantages compared to free drugs, including sustained/ targeted delivery to improve efficacy, and reduction of drug side effects^23–25^. Recently, the Kannan group has shown that postnatal, systemic delivery of poly (amidoamine) dendrimers can target activated microglia and astrocytes in the injured pup’s brain and retina^26, 27^. They showed that a dendrimer-NAC conjugate (DNAC) nanoparticle attenuates microglial activation *in vitro* and decreased the production of tumor necrosis factor alpha (TNF-α), nitric oxide, and peroxides^28^. More importantly, a single systemic dose of DNAC administered after birth to rabbits with CP resulted in marked improvements in motor function, myelination, and attenuation of neuroinflammation^29^. The effect of maternal systemic administration of DNAC on maternal, placental, and fetal response to inflammation has not been explored previously and is the subject of the current study. This may pave the way for preventing/reducing neurodevelopmental disabilities in the newborn.

We have previously used a mouse model of lipopolysaccharide (LPS) - induced intrauterine inflammation to study the effect of maternal immune activation on fetal inflammatory response syndrome (FIRS)^8, 14, 15, 30^. During normal pregnancy, the placenta provides a barrier to prevent the maternal immune system from rejecting the fetus. We hypothesized that DNAC will reduce placental inflammation, prevent preterm birth and will result in a decrease in perinatal brain injury. To our knowledge, this study is the first to use maternal nanoparticle-based therapy to prevent preterm birth and to evaluate placental inflammation and to contrast it to free NAC.

## Results

### Preparation and characterization of dendrimer-NAC conjugate (DNAC)

We successfully prepared, characterized, and validated the DNAC conjugate and the drug release mechanism for DNAC as described previously^24, 29, 31^. In brief, we functionalized hydroxyl - terminated generation-4 poly amidoamino (PAMAM) dendrimer with reactive amine groups using Fmoc protection/deprotection chemistry followed by reaction with NAC using a suitable thiol reactive pyridyldithio propionate linker to get DNAC conjugate. The purity of the conjugate was validated by ^1^H NMR and high-pressure liquid chromatography (HPLC). NIH guidelines for authentication of key chemical compounds was followed. Consistent standards and reproducibility are maintained by authentication for mass payload of NAC, chemical structure, purity, stability and release profile by ^1^H NMR and HPLC as previously described for other *in vivo* studies^29, 31, 32^. Each dendrimer contains 20 molecules of NAC-conjugated, resulting in a drug payload of ~15 wt%. The DNAC conjugate is readily soluble in water, saline and phosphate buffered saline (PBS) (pH 7.4). At physiological conditions, the DNAC conjugate was stable in the absence of GSH over a period of 72 h^24, 29^. At extracellular and plasma GSH levels (2 μM), the conjugate did not release measurable NAC. The NAC was readily released from the conjugates (80% in 100 min) at intracellular GSH concentrations (2 and 10 mM). This indicated that the use of a disulfide linker enabled rapid release of NAC from the conjugate, but only when it was exposed to intracellular GSH-rich environment^24, 29^.

### DNAC reduces preterm birth rate

Mice were monitored for preterm birth for 32 hours after surgery. There were no preterm births among pregnant dams that received intrauterine PBS (n = 12) or intrauterine PBS with DNAC (n = 4) (Table 1). Pregnant dams in the intrauterine LPS group (n = 72) had a preterm birth rate of 75%. Treatment of LPS-exposed dams with DNAC (*n* = 37) significantly reduced the preterm birth rate to 43.2% (p = 0.004, Chi-squared test). In comparison, NAC administration in doses of 10 mg/kg (equivalent to the NAC dose in DNAC) and 100 mg/kg (ten-fold higher NAC dose than that in DNAC) resulted in preterm birth rate of 66.7% (*n* = 12) and 65% (*n* = 20), respectively, which was not significant compared to LPS group. In addition, to evaluate the effect of dendrimer only we injected the same amount of dendrimer as DNAC to LPS model. There was no significant difference between LPS + PBS and LPS + dendrimer (n = 7) on preterm birth rate (Table 1, p > 0.05, Chi-squared test) which is similar to previous study^29^, demonstrating that there was no effect of dendrimer alone since it is an inert vehicle.

**Table 1:** Preterm birth rate.

Treatment	Preterm Birth (%)***
PBS	0/12 (0.0)
LPS	54/72 (75)
PBS + DNAC	0/4 (0)
LPS + DNAC	16/37 (43.2)
LPS + dendrimer	4/7 (57.1)
LPS + NAC10	8/12 (66.7)
LPS + NAC100	13/20 (65.0)

***Chi Square test, p < 0.001.

To see if intrauterine inflammation has effects on postnatal growth of offspring, we weighed the pups at PND 5. At PND5, there was no difference between PBS and LPS groups (p > 0.05, Student’s t-test, and data not shown).

### Dendrimer-Cy5 conjugate localizes to placenta and yolk sac membranes

**Figure 1:**
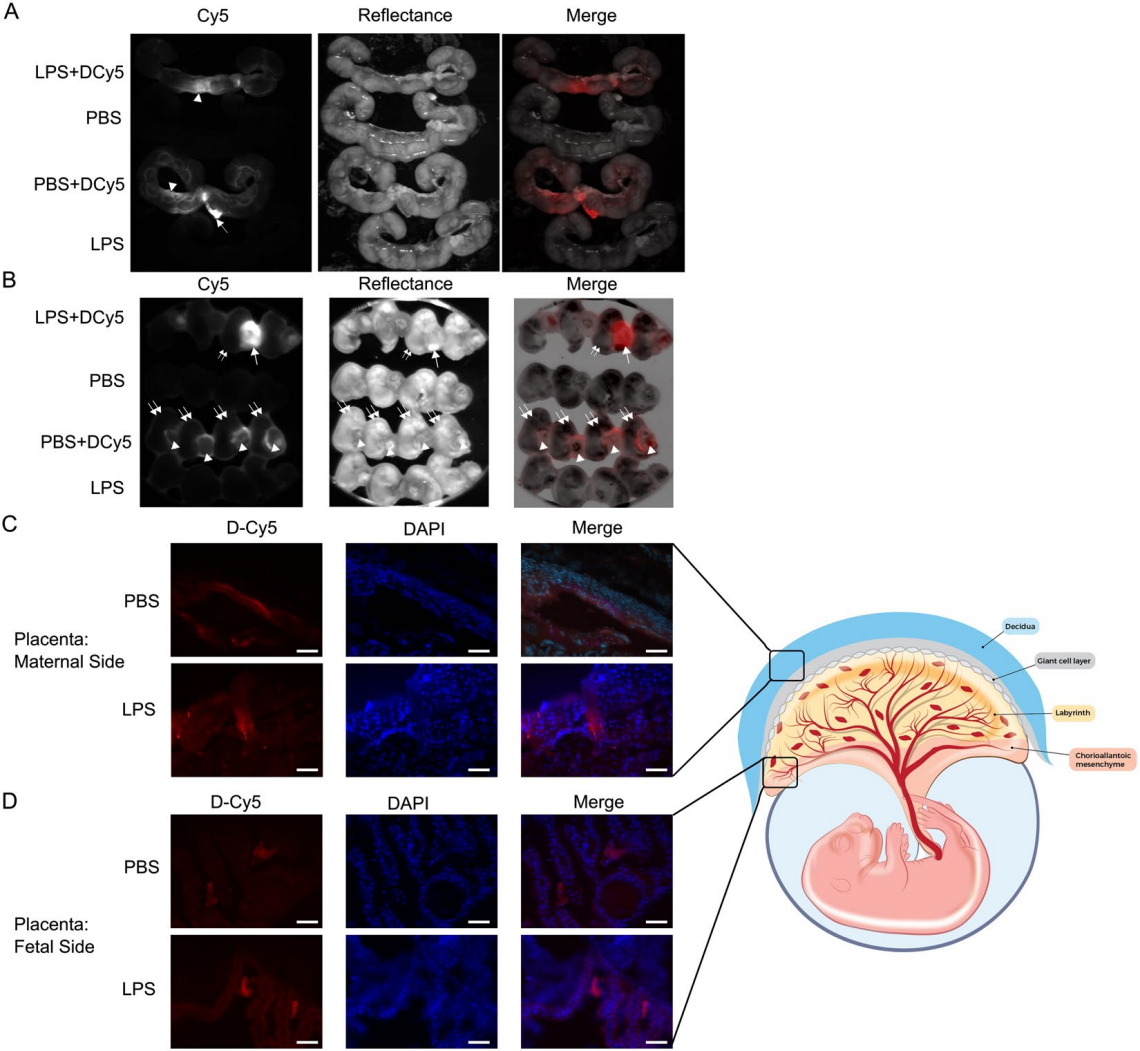
*Ex vivo* fluorescent imaging and immunohistochemistry to monitor trafficking of Cy5-labeled dendrimer (DCy5). On gestational day 17 (E17), pregnant CD-1 dams were given intrauterine injections of LPS or PBS. DCy5 (10 mg/kg) was administered intraperitoneally 1 hour later. After 6 hours, uterus and embryos were isolated, imaged to monitor the fluorescent signal from DCy5, and prepared for histochemical verification. (**A**) The labeled dendrimer accumulated at LPS injected surrounding structure (arrow heads) and in the bladder (arrow). (**B**) Four embryos from each uterus were imaged. The outer embryos are from opposing distal ends of the uterine horns, and the two embryos in the center were taken from either side of the LPS or PBS injection site. The labeled dendrimer was distributed evenly in placentas (arrowheads) from proximal and distal embryos in the PBS group, but had increased accumulation in placentas of embryos near the site of LPS injection (arrow). (**C**) Histochemistry confirmed the presence of DCy5 in the maternal side of the placenta. (**D**) DCy5 was present in the fetal tissue of the placenta, but was restricted to the yolk sac membranes. The presence of DCy5 in the yolk sac increased with LPS exposure. Images are 40× magnifications, and scale bars represent 50 μm. Double arrows: embryo. The diagram was printed with permission © 2016 Jennifer Fairman, CMI, Johns Hopkins University SOM.

To determine the biodistribution of the dendrimer in our model, we utilized dendrimer conjugated with the fluorophore Cy5 (DCy5). Six hours after surgery, *ex vivo* imaging and histochemical assessments were done. DCy5 localized to the uterus and placenta (Fig. 1A). Regardless of whether LPS or PBS was injected, the systemic dendrimer appeared to accumulate near the site of LPS/PBS injection in the uterus (Fig. 1A). DCy5 accumulated more in placentas proximal to the LPS injected surrounding structure (Fig. 1B). In mice injected with PBS, the localization of dendrimer in the placenta was evenly distributed amongst embryos throughout the uterus (Fig. 1B). Confirmatory histochemistry verified that the dendrimer was present in placental tissue (Fig. 1C,D). With PBS injection, the dendrimer was localized primarily in maternal tissues of the placenta (Fig. 1C). We observed stronger signaling in the fetal yolk sac with LPS injection compared to PBS (Fig. 1D).

### DNAC induces regulatory immune response in placenta

**Figure 2:**
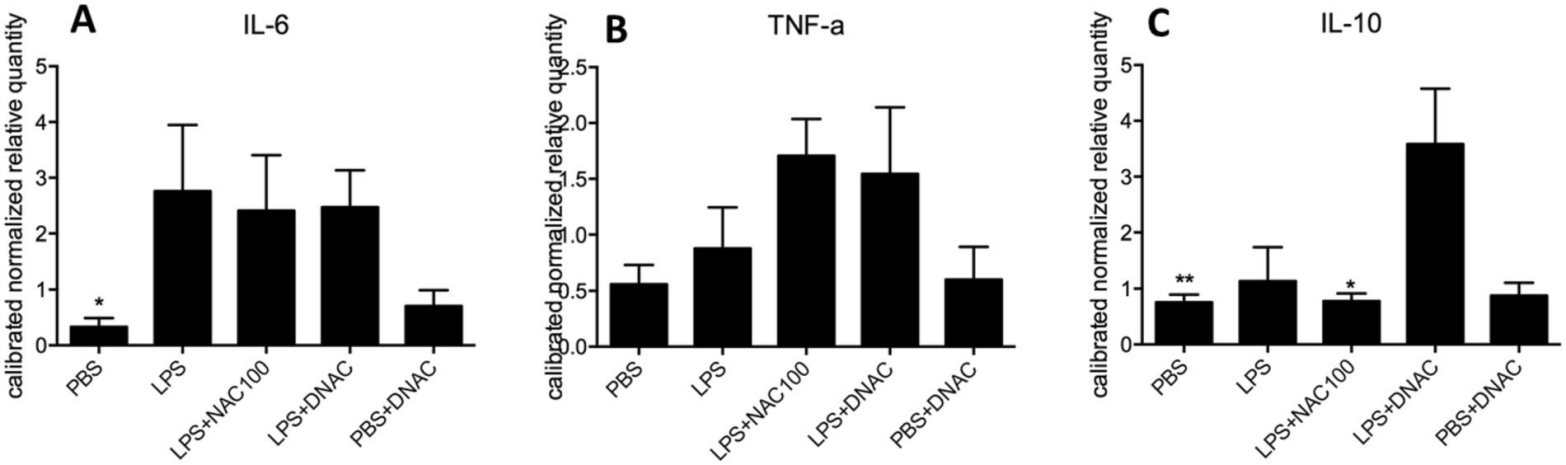
DNAC suppresses inflammatory response in the placenta. cDNA was prepared from RNA isolated from placentas (**A**–**C**) at 6 hours post-surgery, and RT-qPCR was performed to assess cytokine gene expression. Cytokine expression was normalized to 18S ribosomal, β-actin, GAPDH, and HPRT RNA expression. DNAC treatment significantly increased the expression of IL-6 and IL-10 in LPS-exposure group, compared to PBS control (**A** and **C**). There were no significant changes between PBS and PBS + DNAC group for IL-6, TNF – α and IL-10 expression. Data was log transformed for statistical analysis. (*p < 0.05 vs LPS + DNAC, **p < 0.01 vs LPS + DNAC); One-way ANOVA with Newman-Keuls for multiple comparisons; *n* = 6 PBS litters, 3 LPS litters, 5 LPS + DNAC litters, 5 PBS + DNAC litters, 5 LPS + NAC100 litters). GAPDH, Glyceraldehyde 3-phosphate dehydrogenase; HPRT, hypoxanthine phosphoribosyltransferase 1.

We next performed quantitative reverse transcription PCR (RT-qPCR) to measure the expression of interleukin (IL) - 6, TNF-α and IL-10 in the placenta. In placenta, despite the high mRNA levels of pro-inflammatory cytokines, IL-6 and TNF-α in LPS + NAC100 or LPS + DNAC, which were similar to LPS, DNAC significantly increased the regulatory cytokine IL-10 mRNA levels following LPS exposure, compared to PBS and LPS + NAC100 groups (p < 0.01 and p < 0.05, respectively, One-Way ANOVA, Fig. 2) The similar change was not observed with free NAC treatment of 100 mg/kg (Fig. 2). Treatment of DNAC to the surgical control (PBS and surgery) did not show any significant difference from the vehicle administered group.

### DNAC prevents LPS-induced placental CD8^+^ T-cell infiltration

**Figure 3:**
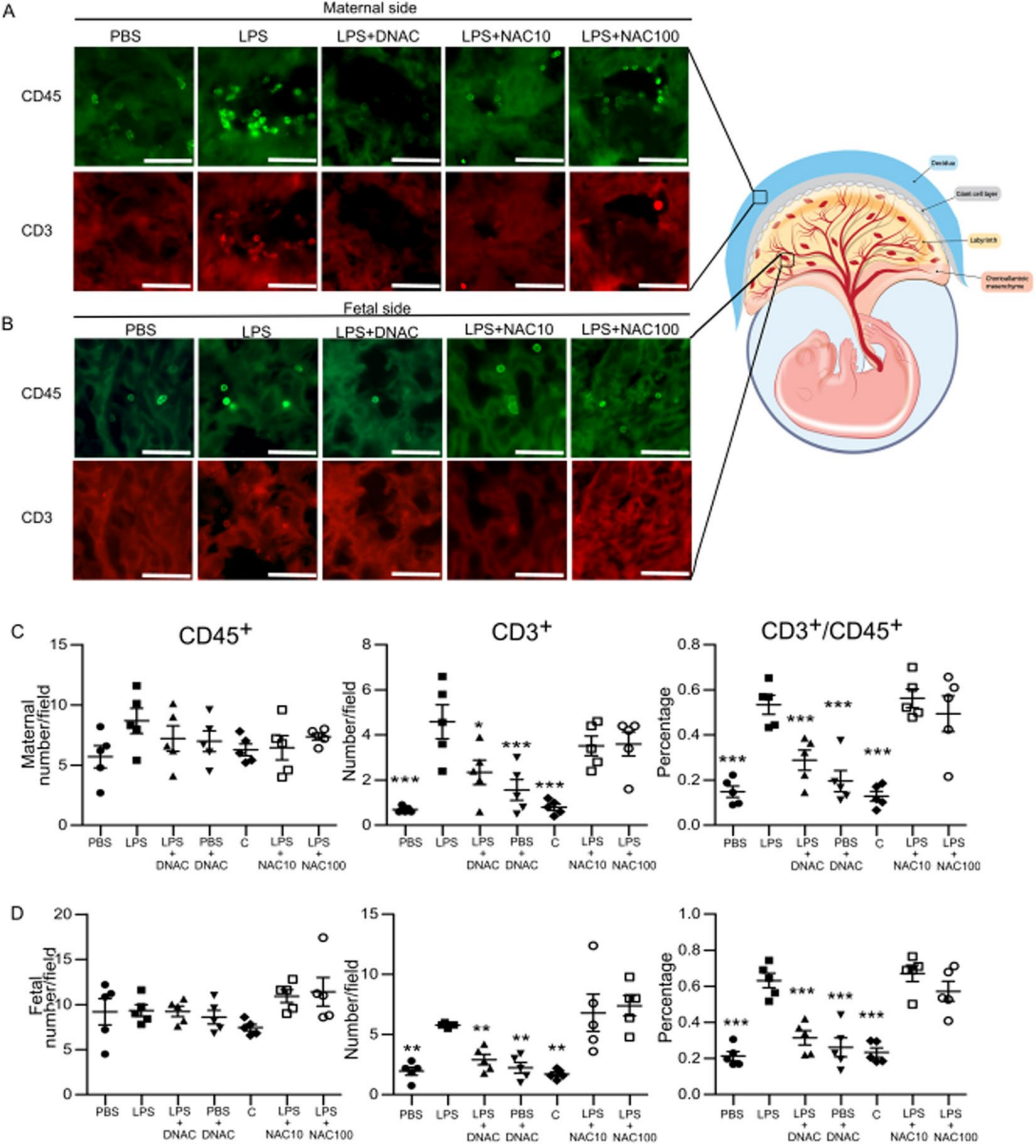
DNAC suppresses CD3^+^ T- cell recruitment to the placenta. (**A**,**B**) Placentas were collected at 6 hours after intrauterine LPS injection and stained with anti-CD45 (a marker for hematopoietic cells) and anti-CD3 (a marker for T cells) antibodies. DAPI staining identified nuclei. CD45^+^ and CD3^+^ cells were quantified in the maternal and fetal sides of the placenta (vasculature excluded). CD3^+^/CD45^+^ cells were observed at the margins of vessel walls in LPS group. This phenomenon was not observed in the other groups evidently. The diagram was printed with permission © 2016 Jennifer Fairman, CMI, Johns Hopkins University SOM. (**C**,**D**) LPS increased CD3^+^ cells and ratio of CD3^+^/CD45^+^ cells in both the maternal and fetal sides of the placenta compared to PBS and DNAC groups (*p < 0.05, **p < 0.01 and ***p < 0.001 vs. LPS; One-Way ANOVA with Bonferonni post-hoc test, *n* = 5 for all groups (7 groups, 35 litters)). Images are 40× magnification, and scale bars represent 50 µm.

Following the observation that regulatory cytokine expression in the placenta was increased by DNAC, we performed immunohistochemistry and flow cytometry to analyze immune cell infiltration. To exclude the vascular leucocytes, double staining of endothelial marker (vimentin) and CD45 on placenta (Supplementary Fig. S1) was performed. At 6 hours after LPS exposure in the placenta, there was an increase in CD3^+^/CD45^+^ T-cells in both the maternal and fetal side of the placenta (Fig. 3A,B). Quantification of placental immune cells showed that DNAC treatment significantly reduced the LPS-induced infiltration of CD3^+^ T-cells (p < 0.01, One-Way ANOVA). Administration of free NAC did not ameliorate the infiltration of CD3^+^/CD45^+^ T-cells in LPS-exposed placentas (Fig. 3C,D).

**Figure 4:**
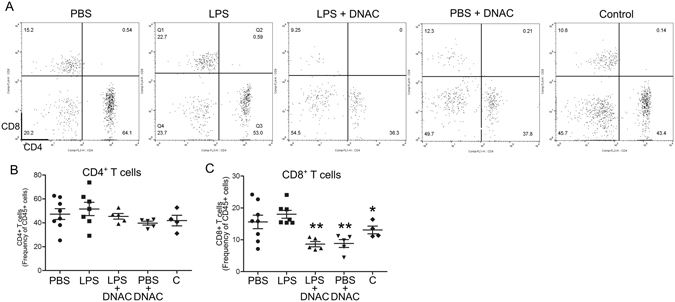
DNAC suppresses CD8^+^ T cell recruitment to the placenta. Single-cell suspensions were prepared and flow cytometry was performed to quantify CD4^+^ and CD8^+^ T cells. (**A**) The frequency of CD4^+^ T cells did not change between the treatment groups (p > 0.05; One-Way ANOVA, *n* = 8 PBS, 7 LPS, 5 LPS + dendrimer *N*-acetyl-*L*-cysteine (DNAC), 5 PBS + DNAC, 4 non-surgical control (Control). (**B**) Treatment with DNAC significantly reduced placental CD8^+^ T cells compared to LPS. (p < 0.01, One-Way ANOVA with Bonferonni post hoc test: *p < 0.05 vs. LPS, **p < 0.01 vs. LPS; *n* = 4 PBS litters, 5 LPS litters, 3 LPS + DNAC litters, 3 PBS + DNAC3 litters, 4 Control litters).

Analysis of CD4^+^ and CD8^+^ subsets by flow cytometry showed that while the frequency of CD4^+^/CD45^+^ cells was not changed between the treatment groups (Fig. 4A,B), maternally-administered DNAC treatment following the exposure to LPS significantly reduced CD8^+^ T-cell frequency versus LPS (p < 0.01, One-Way ANOVA).

### DNAC reduces LPS-induced microglial activation

**Figure 5:**
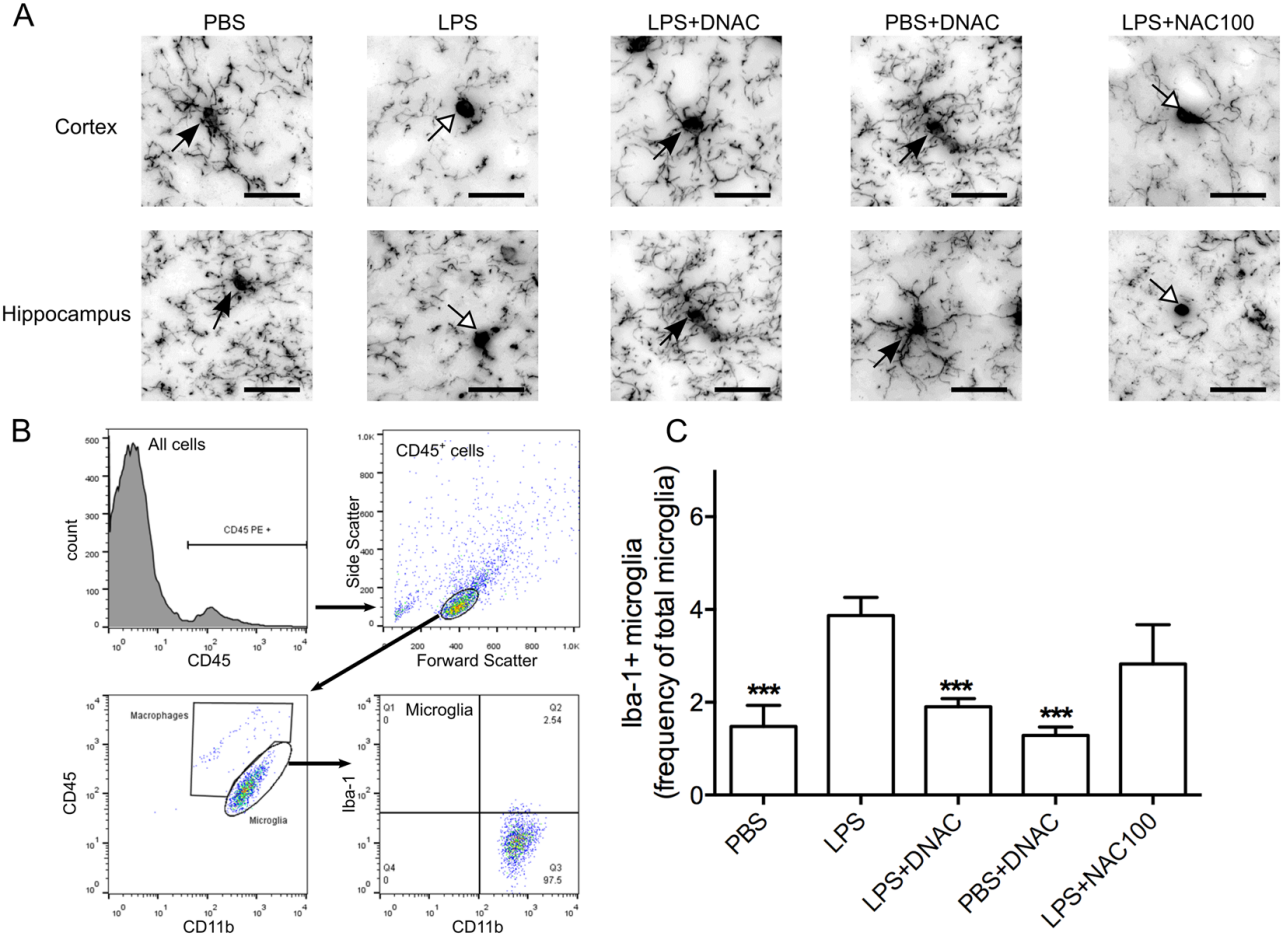
Immunohistochemical staining (IHC) and flow cytometric analysis of postnatal microglial activation at PND17. (**A**) At postnatal day (PND) 17, LPS-exposed offspring had increased numbers of activated microglia (less cell ramification and more amoeboid shape, white arrows) in the cortex and hippocampus compared to PBS and dendrimer *N*-acetyl-*L*-cysteine (DNAC) treatment groups (black arrows). (**B**) Methodology of flow cytometry on microglial activation. Single-cell suspensions were prepared from brains, stained with CD45, CD11b, and Iba-1 antibodies, and analyzed by flow cytometry. Microglia was identified as CD11b^+^/CD45^+^ and Iba-1 expression was assessed to measure microglial activation. (**C**)Based on the methodology of (**B**), LPS-exposed offspring had an increased number of Iba-1^+^ microglia compared to PBS. DNAC treatment resulted in reduced numbers of activated microglia versus LPS alone. (***p < 0.001; One-Way ANOVA; *n* = 6 PBS litters, 9 LPS litters, 5 LPS + DNAC litters, 4 PBS + DNAC litters, 3 LPS + NAC100 litters). Images are 100× magnification, and scale bars represent 5 μm.

To determine the effect of DNAC on microglial activation, we analyzed microglia by immunohistochemistry (ionized calcium binding adaptor molecule 1 (Iba-1) staining) and flow cytometry at PND17. Iba-1 immuno-staining demonstrated an increase in activated microglia morphology (amoeboid shape in cell body and less ramification of cell branches) in LPS-exposed cortex and hippocampus compared to PBS control. DNAC-treated brains showed less microglial activation pattern compared to LPS-exposed and free NAC-treated brains (Fig. 5A).

For flow cytometry analysis, we prepared single-cell suspensions from neonatal brains and identified microglia as CD11b^+^/CD45^+^ cells with a monocyte phenotype based on forward scatter and side scatter (Fig. 5B). LPS-exposed offspring had a significant increase in activated microglia compared to PBS control (p < 0.001, One-Way ANOVA) (Fig. 5C). DNAC treatment did not induce microglia activation in PBS-treated offspring and significantly reduced activated microglia compared to the LPS group. NAC administration showed no significance in preventing the activation of neonatal brains (p < 0.001, One-Way ANOVA) (Fig. 5C).

### DNAC improves neuromotor development

**Figure 6:**
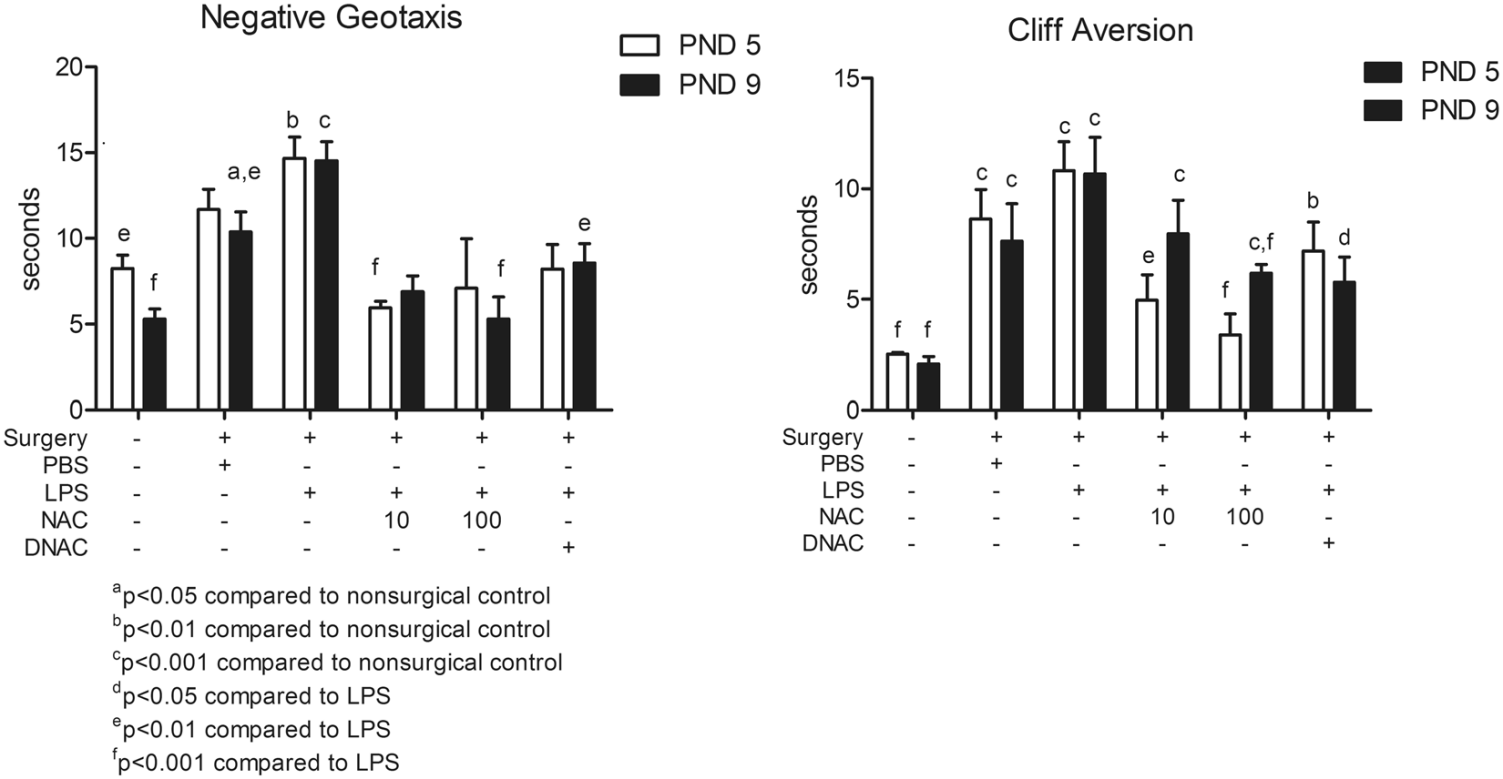
Neuromotor and developmental behavioral changes in response to intrauterine exposure to LPS. Surviving offspring were tested on postnatal day (PND) 5 and 9 to assess neurodevelopmental milestones using negative geotaxis and cliff aversion tests. Prenatal lipopolysaccharide (LPS) exposure induced significant changes in negative geotaxis and cliff aversion test performance in LPS groups at PND 9. Maternal treatment with dendrimer *N*-acetyl-*L*-cysteine (DNAC) significantly improved negative geotaxis behavior at PND 5 and 9, compared to surgery + LPS. Cliff aversion test performance for treated pups was similar to non-surgical control litters at PND9. (Generalized Estimating Equations with pair-wise Bonferroni post-hoc tests; *n* = 18 PBS litters, 27 LPS litters; 17 LPS + DNAC litters; 4 PBS + DNAC litters; 14 LPS + NAC10 litters; 6 LPS + NAC100 litters; 14 Control litters).

We have previously shown that the LPS-induced intrauterine inflammation affects pre-weaning performance on negative geotaxis and cliff aversion tests^10^. In the comparisons, we focused on the effect of our model compared to non-surgical control and the effect of DNAC treatment compared to LPS model. Similar to previous study, LPS-exposed pups exhibited impaired performance compared to PBS controls at postnatal day (PND) 9 on negative geotaxis test (Fig. 6). DNAC and NAC treatment significantly improved negative geotaxis or cliff aversion tests at either PND5 or PND9 (Fig. 6).

## Discussion

For the first time, we demonstrate that maternally-administered prenatal systemic delivery of targeted, dendrimer-conjugated therapy improves perinatal outcomes in a mouse model of intrauterine inflammation, through maternal immunomodulation. Specifically, a single dose of systemic DNAC was found to have a significant impact on prevention of preterm birth and reduction of neuromotor deficits in offspring exposed to *in utero* inflammation, through its direct effects on placenta and indirect effects on fetal brain. At doses of 10 mg/kg, DNAC therapy was more effective than NAC therapy of 100 mg/kg in the prevention of preterm birth and placental and fetal brain immune response. No adverse effects of DNAC were noted in the controls. These findings suggest that DNAC may be an effective therapeutic agent during preterm labor, administered in order to delay the onset of birth and to reduce long-term adverse neurological sequelae in offspring.

Since we already established the component and conjugate safety, we focused this paper on whether ‘maternal administration of D-NAC’ has a positive impact on the fetus and the newborn. The findings reported in this paper are rather significant, and show that even maternal administration of D-NAC may be beneficial, and has significant translational potential, given that D-NAC is undergoing clinical translation for the treatment of newborns and children.

In our prior studies of intra-amniotic administration of dendrimer alone we demonstrated that dendrimers localize in the fetal brain 24 hours after amniotic fluid injection in a rabbit model and mouse model of maternal inflammation^33, 34^. However, human placental perfusion studies using this dendrimer have shown that very little dendrimer crosses the human placenta^35^. In this study, we were not focusing specifically on maternal effects, but were focusing on the fetal/newborn effects of dendrimer-drug therapy administered to the mother. We administered the dendrimer-based therapy one hour after LPS exposure intra-peritoneally (maternal therapy) and harvested tissues six hours after the surgery. Treatment at this time point, primarily affected the placental and maternal response. We used a fluorescent-labeled dendrimer delivered maternally and monitored localization by *ex vivo* imaging and immunohistochemistry. Similar to our previous studies^29, 36–38^, we demonstrated that the dendrimer localizes only in the sites of inflammation, thereby resulting in targeted therapy to placenta. Based on these data, we believe that the responses we see in the fetus and offspring with DNAC therapy are due to its indirect effects on the fetal brain.

Clinically, there are limited options for prevention of preterm birth. Most interventions focus on antibiotics (as in cases of preterm premature rupture of membranes) or short-term tocolytics in order to reduce preterm birth^39^. In most cases, therapy does not address long-term sequalae to the fetus, and it is important to increase therapeutic armamentarium for prevention of progression of preterm labor to preterm birth and inflammation-associated morbidity. NAC has been shown to reduce the rate of recurrent preterm birth in a clinical trial and to decrease preterm birth in animal models if administered before LPS. That being said, a prior study by Xu *et al*.^40^ demonstrated that administering free NAC (total of 300 mg/kg per animal) after LPS exposure led to a higher preterm birth rate compared to no treatment. The doses used by Xu *et al*. were higher than those used in our study (10 mg/kg and 100 mg/kg). This evidence supports our hypothesis that free NAC at various doses (10, 100, and 300 mg/kg) does not reduce preterm birth and may have adverse side effects, and that a superior treatment is low-dose, dendrimer-bound NAC^20–22, 41^. Typical doses on NAC used in other studies for maternal inflammation have used 100–300 mg/kg of NAC systemically every 6 hours^42, 43^. DNAC, a conjugate of NAC with a dendrimer nanoparticle (~4 nm in size), has been synthesized and successfully used in various animal models to prevent adverse neurologic outcomes^29, 31–34^. The dose that we have used here is a 10th of that dose and is administered only once. This will avoid high doses in the fetus and the doses that have reported side effects in human studies. Our study is the first to demonstrate that DNAC is superior in prevention of preterm birth, and in reducing ongoing inflammation and microglial activation after birth. Importantly, treatment of DNAC to the ‘healthy’ surgical control (PBS) mothers did not show significant effect compared to vehicle controls, suggesting that DNAC does not induce an immune response in healthy animals.

Intrauterine inflammation, induced by LPS, leads to toll-like receptor 4 (TLR-4) activation and the production of acute phase cytokines mediated through the NFκB pathway^44^. IL-10 is a regulatory cytokine that is produced in response to inflammation in order to suppress the inflammatory cascade^45, 46^. It plays an important role in further activation and recruitment of immune cells^47–50^. In our studies, we demonstrate that in comparison to free NAC, DNAC is able to increase IL-10 production in placenta (Fig. 2C). We speculate that IL-10 production is one of the key players at the maternal-fetal interphase which assists in maternal immunomodulation and fetal response to intrauterine inflammation.

Immune cells, specifically T cells, play a crucial role in regulating the maternal-fetal interface and its response to inflammation in pregnancy. T cell tolerance and trafficking are regulated by decidual stroma cells in the placenta, providing additional protection from aberrant maternal immunity^51–53^. When challenged with inflammation, the placental barrier function is weakened, allowing T cell infiltration^54^. Indeed, our immunohistochemical results indicate that intrauterine inflammation leads to an increase in CD3^+^/CD45^+^ cells on both maternal and fetal sides of the placenta, with a significant decrease following DNAC but not free NAC treatment (Fig. 3C). Further analysis by flow cytometry confirmed an increase in CD8^+^ cytotoxic T cells following intrauterine inflammation. Treatment with DNAC prevented the increase in CD8^+^ T-cell infiltrates, indicating that trafficking and homing signaling was disrupted (Fig. 4C).

The flow cytometry data represented the whole profiles of CD4 and CD8, demonstrating the decreased numbers, including the intravascular and extravascular CD8 cells by DNAC administration. The IHC data showed the infiltrated T cells (extravascular CD4 and CD8 T cells). This may imply that the infiltrated T cells were CD8 cells. Due to technical limitations, we cannot separate T cells in the blood vessels by flow cytometry, and CD4 or CD8 T cells by IHC.

Others have shown that T lymphocytes are not key players in the regulation of preterm birth^55^. Therefore, we do not attribute DNAC-associated decreases in preterm birth to the reduction of placental T cell infiltrates. As we know from our prior studies, preterm labor, and perinatal brain injury have divergent mechanisms^56^. While CD8^+^ cytotoxic T cells do not play a role in preterm labor, we speculate that, CD8^+^ T cells may be involved the mechanism of LPS-induced intrauterine inflammation. We further speculate that reduction in placental CD8^+^ T cells led to a decrease in placental inflammatory load which indirectly benefited fetal neurodevelopment.

Microglia is tissue-resident macrophages that form the basis of the brain’s immune system for the fetus^57, 58^. They have been shown to be persistently activated into adulthood following *in utero* exposure to inflammation and play a role in adverse neurological outcomes^10^. In our study, we examined the effect of maternally administered DNAC on microglial activation in the presence of intrauterine inflammation. Studies have already demonstrated that microglia contribute to the development of neurons^59, 60^. Postnatally, we focused on the cortex and hippocampus, in which neurons are primarily located. Similarly, we found decreased microglial activation only with DNAC. While preweaning neurobehavior was comparable between DNAC and free NAC following exposure to intrauterine inflammation, we speculate that decreased microglial activation may play a role in long-term postweaning neurological function. As we have previously shown that adult offspring (PND 120) of dams that were exposed to LPS *in utero* exhibit chronic brain inflammation, with persistent microglial activation and aberrant post-weaning behavior, we speculate that DNAC may prevent these prolonged detrimental effects^10^.

In summary, in our study, we provide evidence that a single, maternal systemic DNAC treatment (at 10 mg/kg dose) prevents inflammation-induced preterm birth rate, reduces placental and fetal brain inflammation and improves neurologic outcomes in offspring, at doses that are ten times lower than free NAC. The significance of the study is that, even though the therapy is administered to the mother, without crossing to the fetus, it has a positive impact on the fetus and the newborn. Our results suggest a mechanism by which acute pro-inflammatory signaling is attenuated in the placenta, resulting in a decrease in placental immune activation and a reduction in perinatal brain injury. The acute maternal immune response leads to placental infiltration of peripheral immune cells, suggesting that compromise of the maternal-fetal barrier occurs early in the course of an immune response. Additional work is warranted to clarify the mechanism by which DNAC prevents preterm birth, placental and fetal brain immune activation, and infiltration of CD8^+^ T cells. The positive maternal therapeutic outcomes following a relatively low dose of dendrimer-conjugated NAC (DNAC-10 mg/kg of NAC), combined with the positive safety profile of these dendrimers in the newborn rabbits, offers promise for clinical translation in the perinatal period^29^.

## Methods

### Mouse model of intrauterine inflammation

**Figure 7:**
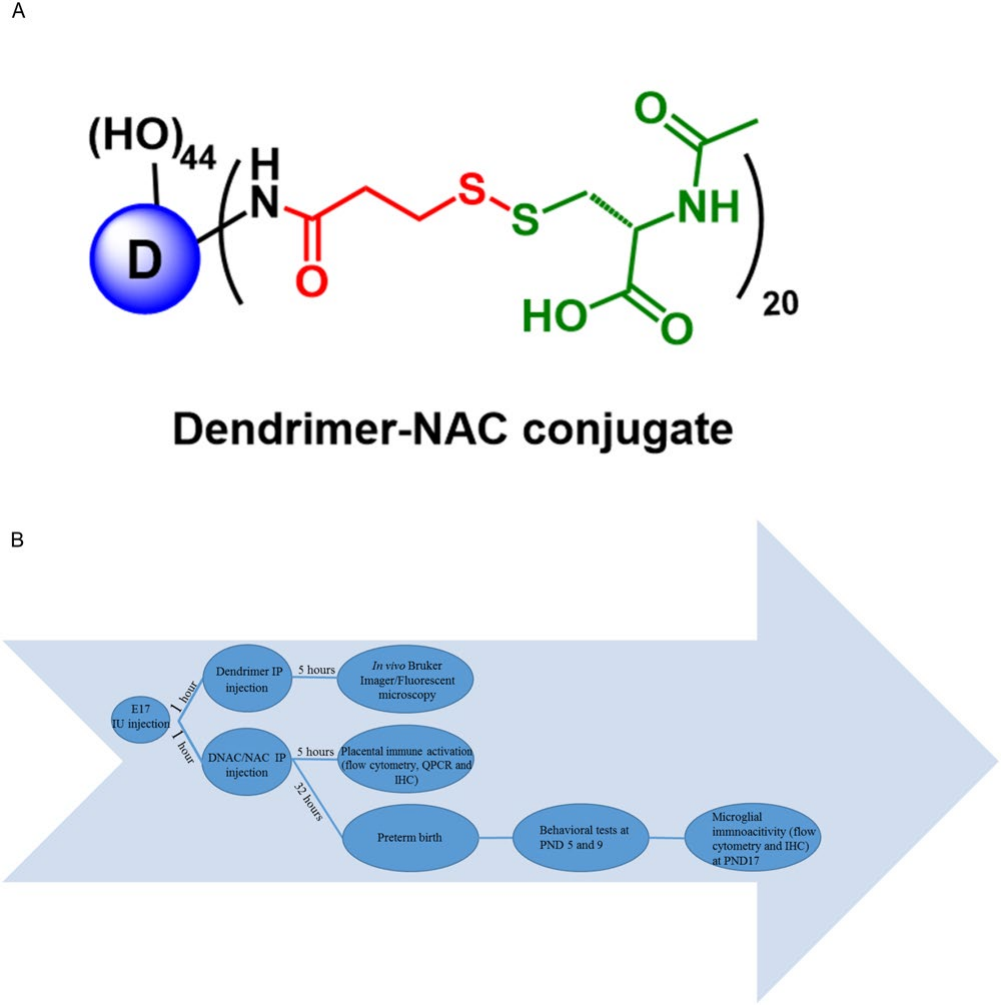
Schematics of dendrimer –NAC and experimental flow. (**A**) Chemical formular of dendrimer- NAC (DNAC). (**B**) Schematic of experimental design. E: embryonic day; IU: intrauterine; IP: intraperitoneal; IHC: immunohistochemistry; PND: postnatal day.

All animal care and treatment procedures were approved by the Animal Care and Use Committee of Johns Hopkins University. All methods were performed in accordance with the relevant guidelines and regulations of Johns Hopkins University. Timed pregnant CD-1 mice were obtained from Charles River Laboratories (Wilmington, MA), and an established model of intrauterine inflammation was used as previously described^8, 10, 13, 14, 30, 61–63^. Briefly, pregnant dams underwent laparotomy at embryonic day 17 (E17) and were injected with either 25 μg LPS (from *E*. *coli* O55:B5; Sigma Aldrich, St Louis, MO) in 100 μl PBS or 100 μl of PBS alone between the first and second embryos of the right uterine horn. The injected structure was inside uterine muscle (intrauterine) between the first and second sac of right horn of murine uterus^60^. At 1 hour after the surgery was performed, dams from LPS-exposed and PBS - exposed groups received via intraperitoneal (IP) injection either 150 μl of DNAC (Fig. 7A, 10 mg/kg), 150 μl of free NAC (10 mg/kg), 150 μl of free NAC (100 mg/kg), Cy5-labeled dendrimer (10 mg/kg), 150 µl PBS, or no intervention (non-surgical controls). As indicted in Fig. 7B, outcomes included preterm birth, placental immune activation at 6 h post-surgery, neurobehavioral at PND 5 and 9 and microglial activation at PND 17. These time points were chosen consistent with previous studies^10, 64^.

### Preterm birth

Mouse gestation is 19–20 days, and we consider all deliveries prior to E19 as preterm birth. Therefore, we observe mice for delivery up to 32 h post-surgery at E17 for any deliveries that would be considered preterm. The preterm birth rate was determined for the following: n = 12 PBS dams, 65 LPS dams, 4 PBS + DNAC dams, 37 LPS + DNAC dams, 20 LPS + NAC100 and 12 LPS + NAC10.

### Behavioral evaluation

A developmental milestone scoring system was used as previously described with modifications^65^. Motor development was assessed by negative geotaxis and cognitive development was measured by cliff aversion on PND 5 and 9. The tests were conducted as follows: *n* = 18 PBS litters, 27 LPS litters; 17 LPS + DNAC litters; 4 PBS + DNAC litters; 14 LPS + NAC10 litters; 6 LPS + NAC100 litters; 14 non-surgical control litters. We have previously shown that both in *in-vitro* and *in-vivo* studies that this neutral hydroxyl dendrimer has no effect on markers of inflammation or neurological function^29, 32^.

### RT-qPCR

We dissected two placentas at right horn of mouse uterus randomly but excluded the first and second sac of each dam and applied pooled RNA extraction for RT-PCR experiment. RNA was prepared using RNeasy Mini Kit (Qiagen, Valencia, CA), and cDNA was synthesized using iScript cDNA synthesis kit (Bio-Rad, Hercules, CA). The following probes were obtained from Integrated DNA Technologies (Coralville, IA): IL-6, Mm.PT.58.10005566; IL-10, Mm.PT.58.13531087; TNF-α, Mm.PT.58.29509614; glyceraldehyde 3-phosphate dehydrogenase (GAPDH), Mm PT. 39a.1; hypoxanthine-guanine phosphoribosyltransferase (HPRT), Mm. PT. 39a.22214828 and β-actin, Mm.PT.58.33540333. 18S ribosomal RNA endogenous control primers were obtained from Life Technologies (Grand Island, NY). RT-qPCR was performed with Universal Master Mix II (Life technologies, Frederick, MD) on a CFX384 Real-Time PCR Detection system (Bio-Rad, Hercules, CA). The RNA was isolated from placentas at 6 h post-surgery for the following groups: *n* = 6 PBS litters, 3 LPS 3 litters, 5 LPS + DNAC litters, 5 PBS + DNAC litters, 5 LPS + NAC100 litters.

### Immunohistochemistry and *ex vivo* imaging

Placenta and fetal brain were harvested 6 h following surgery as well as PND 17 brains for examination of pre-weaning brain anatomy. Using a cryostat (Leica; Buffalo Grove, IL), placenta, fetal and postnatal brains were sectioned into 20 μm thick slices and then attached to Superfrost Plus Microscope Slides (Fisher; Jessup, MD), followed by drying at room temperature. Sections were incubated overnight at 4 °C with primary antibodies in PBS containing 0.5% Triton X-100 (Sigma-Aldrich, St. Louis, MO) and 3% horse serum (Life Technologies, Frederick, MD). The placenta was stained with rat anti-CD45 (BD Pharmingen, San Jose, CA), rabbit anti-CD3 (DAKO, Carpinteria, CA) and rabbit anti-vimetin (Abcam, Cambridge, MA). CD45 is a leucocyte marker and CD3 is a T cell marker. Vimetin is an endothelial cell marker. Fetal and postnatal brains were stained with rabbit anti- Iba-1 (Wako, 019–19741, Richmond, VA). Iba-1 is a microglia/macrophage-specific calcium-binding protein and identifies activated microglia^66^. The next day, sections were rinsed with PBS, and then incubated with fluorescent secondary antibodies diluted 1:500 for 3 hours at room temperature. The following antibodies were used for immunofluorescence: goat anti-rabbit DyLight 568 (Abcam, Cambridge, MA) and donkey anti-rat Alexa Fluor 488 (Life Technologies, Grand Island, NY). The sections were further stained with DAPI (Roche, Indianapolis, IN) for 2 min at room temperature followed by mounting with Fluromount-G (eBioscience, San Diego, CA). Images were obtained using an Axioplan 2 Imaging system (Carl Zeiss, Thornwood, NY) from the same staining batch. The area of field was chosen as indicated by the diagram (Fig. 3), avoiding the edge of tissue, tissue folds and any other artifacts. Cell count was performed by Image J 1.37 V (NIH) on 10 chosen fields in placenta per animal (n = 5 per group, 7 groups). In this study, the maternal side was referred to the decidua layer of placenta and fetal side was referred to the labyrinth layer and yolk sac. Two independent observers performed the cell quantification.

*Ex vivo* imaging was performed at 6 h after surgery using an MS FX PRO *in vivo* imager (Bruker, Billerica, MA). For Cy5, a 630 nm excitation filter and 730 nm emission filter was used. Reflectance images were acquired without filters.

### Flow cytometry

Cortex from mouse brain was homogenized and digested using Neural Tissue Dissociation Kit (T) (Miltenyi, San Diego, CA) according to the manufacturer’s protocol. Briefly, brains were removed and washed in PBS, placed on a petri dish and cut in fragments and transferred into the gentlimax Tube containing 1950 μL of enzyme mix 1 plus 30 μL of enzyme mix 2. Single-cell suspensions were adjusted to 30% Percoll in HBSS and centrifuged at 300 g for 10 minutes to remove myelin. Cell pellets were washed in HBSS and resuspended in FACS Buffer (2% FBS in PBS) for staining. Placentas were collected at 6 hours after LPS exposure, digested with collagenase Type IV (Sigma). Single cells suspensions were labeled with the following mAbs: anti-CD45-PE conjugated (BD, clone 30-F11); anti-CD8- pacific orange conjugated (Life technologies, clone 5H10), anti-CD4- PercP Cy 5.5 conjugated (BioLegend, clone RM4–4), Iba-1- FITC conjugated (Abcam, clone:1022–5); CD11b - PercP Cy 5.5 conjugated (BD, clone M1/70) diluted 1:50–1:100 in FACS buffer for 30 minutes at 4 °C in the dark, and analyzed on a BD FACSCalibur (Becton Dickinson, Franklin Lakes, NJ). Analysis of flow cytometry data was performed using FlowJo (Tree Star, Ashland, OR).

### Statistics

Data analyses were performed with Prism 5 (GraphPad Software, Inc, La Jolla, CA) or SPSS (IBM Corp., Armonk, New York). Preterm birth rate data were analyzed using Chi-square test. RT- qPCR gene expression and immunohistochemistry data were analyzed using One-Way ANOVA with Bonferonni post-hoc test for multiple comparisons of normally-distributed data and Kruskal-Wallis with Dunn’s multiple comparisons for non-parametric data. Nested litter behavior data was analyzed using Generalized Estimating Equations with Bonferroni pair-wise comparisons in SPSS.

## Electronic supplementary material

Supplementary Information

## References

[1] Allin M (2001). Cognitive and motor function and the size of the cerebellum in adolescents born very pre-term. Brain.

[2] Dammann O, Leviton A (1997). Maternal intrauterine infection, cytokines, and brain damage in the preterm newborn. Pediatr Res.

[3] Edgin JO (2008). Executive functioning in preschool children born very preterm: relationship with early white matter pathology. J Int Neuropsychol Soc.

[4] Goldenberg RL, Culhane JF, Iams JD, Romero R (2008). Epidemiology and causes of preterm birth. Lancet.

[5] Woodward LJ, Edgin JO, Thompson D, Inder TE (2005). Object working memory deficits predicted by early brain injury and development in the preterm infant. Brain.

[6] Yoon BH (2000). Fetal exposure to an intra-amniotic inflammation and the development of cerebral palsy at the age of three years. Am J Obstet Gynecol.

[7] Rees S, Harding R, Walker D (2011). The biological basis of injury and neuroprotection in the fetal and neonatal brain. Int J Dev Neurosci.

[8] Elovitz MA (2011). Intrauterine inflammation, insufficient to induce parturition, still evokes fetal and neonatal brain injury. Int J Dev Neurosci.

[9] Angelidou A (2012). Perinatal stress, brain inflammation and risk of autism-review and proposal. BMC Pediatr.

[10] Dada T (2014). Mouse model of intrauterine inflammation: sex-specific differences in long-term neurologic and immune sequelae. Brain Behav Immun.

[11] Johnston MV, Trescher WH, Ishida A, Nakajima W (2000). Novel treatments after experimental brain injury. Semin Neonatol.

[12] Gayle DA (2004). Maternal LPS induces cytokines in the amniotic fluid and corticotropin releasing hormone in the fetal rat brain. Am J Physiol Regul Integr Comp Physiol.

[13] Burd I (2009). Beyond white matter damage: fetal neuronal injury in a mouse model of preterm birth. Am J Obstet Gynecol.

[14] Burd I (2010). Inflammation-induced preterm birth alters neuronal morphology in the mouse fetal brain. J Neurosci Res.

[15] Breen K (2012). TLR-4-dependent and -independent mechanisms of fetal brain injury in the setting of preterm birth. Reprod Sci.

[16] Leviton A (2013). Systemic inflammation, intraventricular hemorrhage, and white matter injury. J Child Neurol.

[17] Supramaniam V (2013). Microglia activation in the extremely preterm human brain. Pediatr Res.

[18] Awad N (2011). N-acetyl-cysteine (NAC) attenuates LPS-induced maternal and amniotic fluid oxidative stress and inflammatory responses in the preterm gestation. Am J Obstet Gynecol.

[19] Beloosesky R (2006). N-acetyl-cysteine suppresses amniotic fluid and placenta inflammatory cytokine responses to lipopolysaccharide in rats. Am J Obstet Gynecol.

[20] Shahin AY, Hassanin IM, Ismail AM, Kruessel JS, Hirchenhain J (2009). Effect of oral N-acetyl cysteine on recurrent preterm labor following treatment for bacterial vaginosis. Int J Gynaecol Obstet.

[21] Buhimschi IA, Buhimschi CS, Weiner CP (2003). Protective effect of N-acetylcysteine against fetal death and preterm labor induced by maternal inflammation. Am J Obstet Gynecol.

[22] Beloosesky R, Weiner Z, Ginsberg Y, Ross MG (2012). Maternal N-acetyl-cysteine (NAC) protects the rat fetal brain from inflammatory cytokine responses to lipopolysaccharide (LPS). J Matern Fetal Neonatal Med.

[23] Lee CC, MacKay JA, Frechet JM, Szoka FC (2005). Designing dendrimers for biological applications. Nat Biotechnol.

[24] Kurtoglu YE (2009). Poly(amidoamine) dendrimer-drug conjugates with disulfide linkages for intracellular drug delivery. Biomaterials.

[25] Kannan RM, Nance E, Kannan S, Tomalia DA (2014). Emerging concepts in dendrimer-based nanomedicine: from design principles to clinical applications. J Intern Med.

[26] Yang H (2010). Nanoparticle-mediated brain-specific drug delivery, imaging, and diagnosis. Pharm Res.

[27] Jain NK, Mishra V, Mehra NK (2013). Targeted drug delivery to macrophages. Expert Opin Drug Deliv.

[28] Wang B, Navath RS, Romero R, Kannan S, Kannan R (2009). Anti-inflammatory and anti-oxidant activity of anionic dendrimer-N-acetyl cysteine conjugates in activated microglial cells. Int J Pharm.

[29] Kannan, S. *et al*. Dendrimer-based postnatal therapy for neuroinflammation and cerebral palsy in a rabbit model. *Sci Transl Med* **4**, 130ra146 (2012). 10.1126/scitranslmed.3003162 PMC3492056 22517883

[30] Burd I, Brown A, Gonzalez JM, Chai J, Elovitz MA (2011). A mouse model of term chorioamnionitis: unraveling causes of adverse neurological outcomes. Reprod Sci.

[31] Mishra MK (2014). Dendrimer brain uptake and targeted therapy for brain injury in a large animal model of hypothermic circulatory arrest. ACS Nano.

[32] Nance E (2015). Systemic dendrimer-drug treatment of ischemia-induced neonatal white matter injury. J Control Release.

[33] Burd I (2014). Fetal uptake of intra-amniotically delivered dendrimers in a mouse model of intrauterine inflammation and preterm birth. Nanomedicine.

[34] Zhang F (2016). Surface functionality affects the biodistribution and microglia-targeting of intra-amniotically delivered dendrimers. J Control Release.

[35] Menjoge AR (2011). Transfer of PAMAM dendrimers across human placenta: prospects of its use as drug carrier during pregnancy. J. Control Release.

[36] Grimm JC (2016). Nanotechnology Approaches to Targeting Inflammation and Excitotoxicity in a Canine Model of Hypothermic Circulatory Arrest-Induced Brain Injury. Ann Thorac Surg..

[37] Guo Y (2016). Dendrimers Target the Ischemic Lesion in Rodent and Primate Models of Nonarteritic Anterior Ischemic Optic Neuropathy. PLoS One.

[38] Kambhampati SP (2015). Systemic and Intravitreal Delivery of Dendrimers to Activated Microglia/Macrophage in Ischemia/Reperfusion Mouse Retina. Invest Ophthalmol Vis Sci.

[39] Johnson CT, Farzin A, Burd I (2014). Current management and long-term outcomes following chorioamnionitis. Obstet Gynecol Clin North Am.

[40] Xu DX (2005). Effect of N-acetylcysteine on lipopolysaccharide-induced intra-uterine fetal death and intra-uterine growth retardation in mice. Toxicol Sci.

[41] Wiest DB (2014). Antenatal pharmacokinetics and placental transfer of N-acetylcysteine in chorioamnionitis for fetal neuroprotection. J Pediatr.

[42] Beloosesky R (2013). Prophylactic maternal N-acetylcysteine in rats prevents maternal inflammation-induced offspring cerebral injury shown on magnetic resonance imaging. Am J Obstet Gyneol.

[43] Jenkins DD (2016). Fetal and Neonatal Effects of N-Acetylcysteine When Used for Neuroprotection in Maternal Chorioamnionitis. J Pediatr..

[44] Doyle SL, O’Neill LA (2006). Toll-like receptors: from the discovery of NFkappaB to new insights into transcriptional regulations in innate immunity. Biochem Pharmacol.

[45] Prins JR (2015). Unstable Foxp3+ regulatory T cells and altered dendritic cells are associated with lipopolysaccharide-induced fetal loss in pregnant interleukin 10-deficient mice. Biol Reprod.

[46] Kole A, Maloy KJ (2014). Control of intestinal inflammation by interleukin-10. Curr Top Microbiol Immunol.

[47] Tze LE (2011). CD83 increases MHC II and CD86 on dendritic cells by opposing IL-10-driven MARCH1-mediated ubiquitination and degradation. J Exp Med.

[48] Thibodeau J (2008). Interleukin-10-induced MARCH1 mediates intracellular sequestration of MHC class II in monocytes. Eur J Immunol.

[49] Ishida H, Hastings R, Thompson-Snipes L, Howard M (1993). Modified immunological status of anti-IL-10 treated mice. Cell Immunol.

[50] Moore KW, de W Malefyt R, Coffman RL, O’Garra A (2001). Interleukin-10 and the interleukin-10 receptor. Annu Rev Immunol.

[51] Nancy P, Erlebacher A (2014). T cell behavior at the maternal-fetal interface. Int J Dev Biol.

[52] Nancy P (2012). Chemokine gene silencing in decidual stromal cells limits T cell access to the maternal-fetal interface. Science.

[53] Tay CS, Tagliani E, Collins MK, Erlebacher A (2013). Cis-acting pathways selectively enforce the non-immunogenicity of shed placental antigen for maternal CD8 T cells. PLoS One.

[54] Tossetta G (2014). IL-1beta and TGF-beta weaken the placental barrier through destruction of tight junctions: an *in vivo* and *in vitro* study. Placenta.

[55] Bizargity P, Del Rio R, Phillippe M, Teuscher C, Bonney EA (2009). Resistance to lipopolysaccharide-induced preterm delivery mediated by regulatory T cell function in mice. Biol Reprod.

[56] Leitner K (2014). IL-1 receptor blockade prevents fetal cortical brain injury but not preterm birth in a mouse model of inflammation-induced preterm birth and perinatal brain injury. Am J Reprod Immunol.

[57] Nayak D, Roth TL, McGavern DB (2014). Microglia development and function. Annu Rev Immunol.

[58] Ginhoux F (2010). Fate mapping analysis reveals that adult microglia derive from primitive macrophages. Science.

[59] Shigemoto-Mogami Y, Hoshikawa K, Goldman JE, Sekino Y, Sato K (2014). Microglia enhance neurogenesis and oligodendrogenesis in the early postnatal subventricular zone. J Neurosci.

[60] Cunningham CL, Martinez-Cerdeno V, Noctor SC (2013). Microglia regulate the number of neural precursor cells in the developing cerebral cortex. J Neurosci.

[61] Elovitz MA, Wang Z, Chien EK, Rychlik DF, Phillippe M (2003). A new model for inflammation-induced preterm birth: the role of platelet-activating factor and Toll-like receptor-4. Am J Pathol.

[62] Burd I, Breen K, Friedman A, Chai J, Elovitz MA (2010). Magnesium sulfate reduces inflammation-associated brain injury in fetal mice. Am J Obstet Gynecol.

[63] Burd I, Balakrishnan B, Kannan S (2012). Models of fetal brain injury, intrauterine inflammation, and preterm birth. Am J Reprod Immunol.

[64] Lei J (2015). Murine model: maternal administration of stem cells for prevention of prematurity. Am J Obstet Gynecol.

[65] Hill, J. M., L. M. & Stone, M. M. In *Neuropeptide Techniques*, Neuromethods, ed *Gozes I* Vol. **39** 131–149 (Humana Press, Inc, Totowa, NJ, 2007).

[66] Nakamura R (2013). Availability of a microglia and macrophage marker, iba-1, for differential diagnosis of spontaneous malignant reticuloses from astrocytomas in rats. J Toxicol Pathol.

