# Supplementary material for: Maternal dendrimer-based therapy for inflammation-induced preterm birth and perinatal brain injury
Source: Sci Rep. 2017 Jul 21;7:6106. doi: 10.1038/s41598-017-06113-2 (PMC5522481; doi:10.1038/s41598-017-06113-2)
Supplement: Supplementary file 1 — Supplementary Information [file 41598_2017_6113_MOESM1_ESM.pdf]

Maternal dendrimer-based therapy for inflammation-induced preterm birth and perinatal brain injury

Jun Lei[1], Jason M. Rosenzweig[1], Manoj K. Mishra[2], Wael Alshehri[1], Flavia Brancusi[1], Mike McLane[1], Ahmad Almalki[1], Rudhab Bahabry[1], Hattan Arif[1], Rayyan Rozzah[1], Ghada Alyousif[1], Yahya Shabi[1], Nader Alhehaily[1], Wenyu Zhong[1], Andrea Facciabene[3], Sujatha Kannan[4], Rangaramanujam M. Kannan[2*], Irina Burd[1,5,6*]

[1]Integrated Research Center for Fetal Medicine, Johns Hopkins University School of Medicine, Baltimore, MD, USA, [2]Center for Nanomedicine, Johns Hopkins University School of Medicine, Baltimore, MD, USA, [3]Department of Obstetrics and Gynecology, University of Pennsylvania, Philadelphia, PA, USA, [4]Anesthesiology and Critical Care Medicine, Johns Hopkins University School of Medicine, Baltimore, MD, USA, [5]Department of Neuroscience, Kennedy Krieger Institute, Baltimore, MD USA, [6]Department of Neurology, Johns Hopkins University School of Medicine, Baltimore MD, USA

[*]Corresponding authors:

Irina Burd, M.D., Ph.D.

Department of Gynecology and Obstetrics

Integrated Research Center for Fetal Medicine

Johns Hopkins University School of Medicine

600 North Wolfe Street, Phipps 228

Baltimore, MD 21287, USA

Email: iburd@jhmi.edu

Rangaramanujam M. Kannan, Ph.D.

Department of Ophthalmology/Wilmer Eye Institute

Center for Nanomedicine

Johns Hopkins School of Medicine

400 North Broadway, Smith 6023

Baltimore, MD 21287

Email: krangar1@jhmi.edu

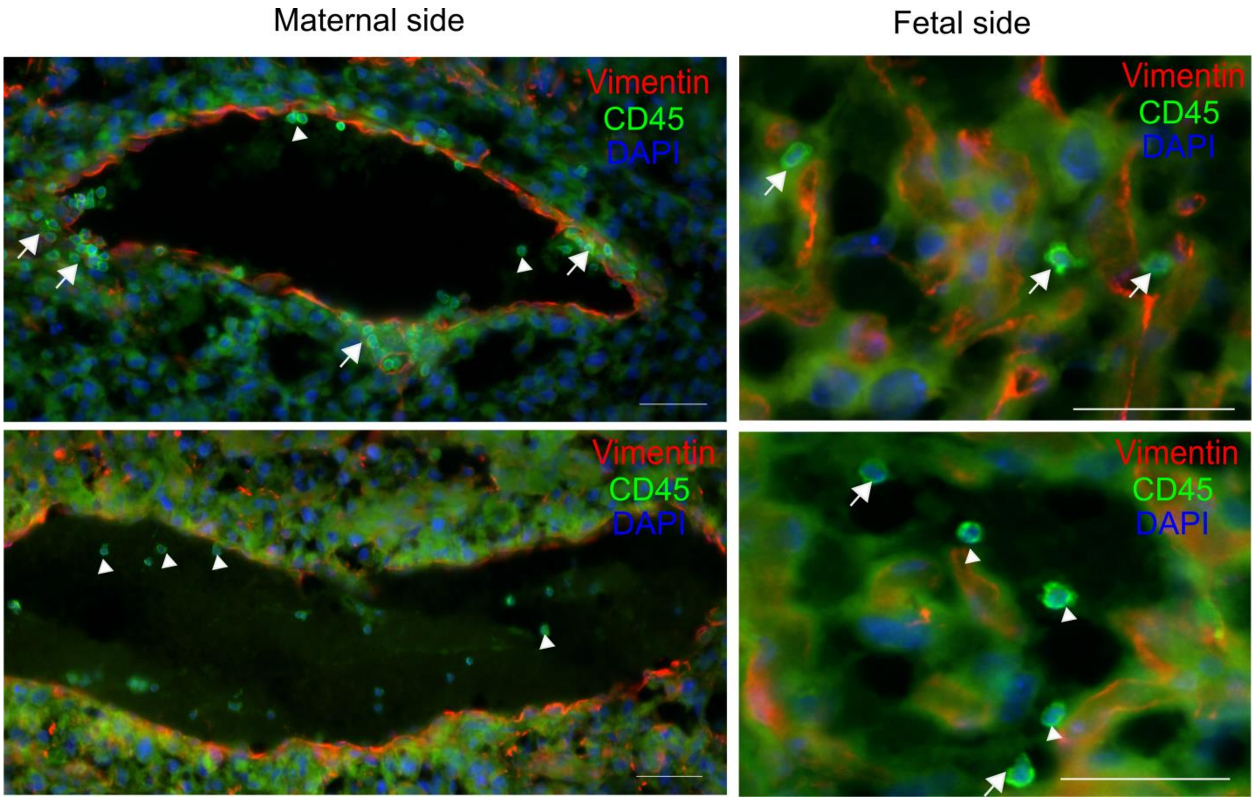

Supplementary Figure S1. Immunohistochemical staining of leucocytes in placenta to distinguish extravascular and intravascular leucocytes. Placenta were collected 6 h after surgery and stained with vimetin (endothelial cell marker), CD45 (leucocytes) and DAPI (nuclear and chromosome counterstain). Left panels were representative images of the maternal side of placenta, abundant in maternal vessels. Right panels were representative images of the fetal side of placenta, major in labyrinth structure. The extravascular leucocytes (white arrows) and intravascular leucocytes (white arrowheads) were indicated. In addition, DAPI staining also helped to exclude the leucocytes in the blood vessel as the red blood cells are DAPI negative. Scale bar: 50 μm.
